# Supplementary material for: Analyses of Molecular Characteristics and Enzymatic Activities of Ovine HSD17B3
Source: Animals (Basel). 2021 Sep 30;11(10):2876. doi: 10.3390/ani11102876 (PMC8532638; doi:10.3390/ani11102876)
Supplement: Supplementary file 1 [file animals-11-02876-s001.zip › animals-1351408-supplementary.pdf]

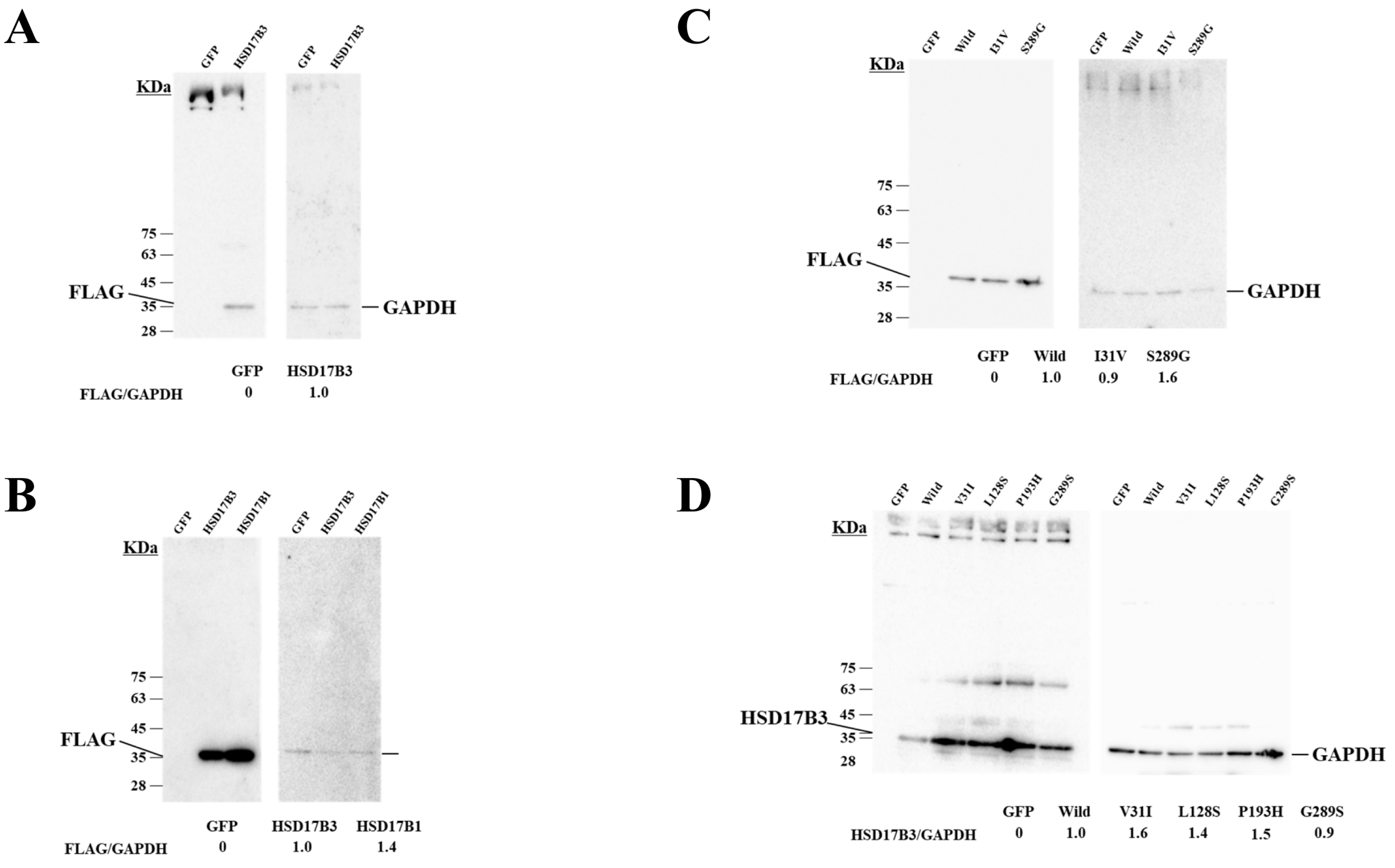

Figure S1 The whole western blot of Fig 2C (A), Fig. 3A (B), Fig. 4C (C) and Fig. 4D (D). The ratio of intensities of signal quantified by densitometry is presented to the bottom. Values of HSD17B3 or wild type HSD17B3 were defined as 1.

```
                                                CCAGGTCTGAAACAGCATCTGCCGTGGTGCCATC
  1 ATGGAGGAGGTTCTGGAGCAGTTCTTCATTTTTGTAGGGTTGCTG
     M  E  E  V  L  E  Q  F  F  I  F  V  G  L  L
 46 GTTTGCCTGGTCTACCTGACGAAATGCGTGAGATTCTCTAAATGC
     V  C  L  V  Y  L  T  K  C  V  R  F  S  K  C
 91 ATTTTCCTGCACTTCTGGAAAGTTTTGCCAAGATCTTTCTTGAAG
     I  F  L  H  F  W  K  V  L  P  R  S  F  L  K
136 TCCATGGGCGAGTGGGCAGTGATCACTGGAGCAGGAGATGGGATT
     S  M  G  E  W  A  V  I  T  G  A  G  D  G  I
181 GGGAAAGCTTACTCATTCGAGTTAGCAAGACAGGGATTCAATGTC
     G  K  A  Y  S  F  E  L  A  R  Q  G  F  N  V
226 GTGCTTATCAGCCGGACACTGGAAAAACTTCAGGCCATTGCTGCG
     V  L  I  S  R  T  L  E  K  L  Q  A  I  A  A
271 GAGATTGAGTGGACTATAGGAAGTACTGTGAAGATAATACAAGCA
     E  I  E  W  T  I  G  S  T  V  K  I  I  Q  A
316 GATTTTGCCAAAGATGACATCTACGAGTATATTAAAGAAAAACTT
     D  F  A  K  D  D  I  Y  E  Y  I  K  E  K  L
361 AAAGGCTTAGAAATTGGAATTTTAGTCAACAATGTTGGAATGCTT
     K  G  L  E  I  G  I  L  V  N  N  V  G  M  L
406 CCAAACCTTCTCCCAAGCCATTTCCTTAACACGCCAGATGACTTC
     P  N  L  L  P  S  H  F  L  N  T  P  D  D  F
451 CAGAGCCTCATCCACTGTAACATCACCTCAGTTGTGAAGATGACA
     Q  S  L  I  H  C  N  I  T  S  V  V  K  M  T
496 CAGCTGATTCTGAAACACATGAAATCAAGGCAGAAAGGTCTCATC
     Q  L  I  L  K  H  M  K  S  R  Q  K  G  L  I
541 TTGAACATATCTTCCGGGGCGGCCCTCTTTCCCTGGCCTCTGTAC
     L  N  I  S  S  G  A  A  L  F  P  W  P  L  Y
586 TCCACGTATTCAGCTTCCAAGGCTTTTGTGTACACATTTTCCAAG
     S  T  Y  S  A  S  K  A  F  V  Y  T  F  S  K
631 GCACTGCAAGCAGAATATAAGGAGAAGGGAATCATCATCCAGGTG
     A  L  Q  A  E  Y  K  E  K  G  I  I  I  Q  V
676 TTGACCCCATATGCTATTTCAACTCCGATGACAAAGTGTCTAAAT
     L  T  P  Y  A  I  S  T  P  M  T  K  C  L  N
721 ACCAACATGATAACCAAGACTGCTGATGAGTTTGTTAAAGAATCA
     T  N  M  I  T  K  T  A  D  E  F  V  K  E  S
766 CTGAATTATGTCACGATTGGAGACGAAACCTGTGGCTGCCTCACC
     L  N  Y  V  T  I  G  D  E  T  C  G  C  L  T
811 CATGAAATCTTGGCTACCATTCTAAGCCTGATCCCATCGTGGGCC
     H  E  I  L  A  T  I  L  S  L  I  P  S  W  A
856 TTCTACAGCAGTGTGTTTCAGAAGATGCTGCTGACTCGTTACGTG
     F  Y  S  S  V  F  Q  K  M  L  L  T  R  Y  V
901 GACTACCTCAAGAAGAATGCCAACATCAGATAG TGCTGGTGAACT
     D  Y  L  K  K  N  A  N  I  R  *

    CGTGTGCCATCCAGCATTGCTTTCCTCACCAGATTCTGCGTTGGCCA
    CAGAGGACACAGGAACAGACCAGTACCTTTTACTTCCCTGAAACTG
    GAG
```

Figure S2 Nucleotide and deduced amino acid sequence of ovine HSD17B3. Positions of nucleotide sequences are indicated on left side. *Stop codon. Primers used in RT-PCR analyses are shown by the boxes. Shaded nucleotides are primers used for cloning. Amino acids different with the data of XM_042243038 are indicated by the circles.

Table S1. Primers used in each experiment.

**PCR primers**	**Forward primers**	**Reverse primers**
**RT-PCR**		
ovine HSD17B3	F- ctcccaagccatttccttaac	R- tcagcagcatcttctgaaaca
ovine GAPDH	F- gtgatgctggtgctgagtac	R- gtagaagagtgagtgtcgc
**cloning**		
ovine HSD17B3	F- ccaggtctgaaacagcatctgc	R- ctccagtttcagggaagtaaaggta

Table S2. HSD17B3/Hsd17b3 nucleotide and deduced amino acid identities in ovine compared with other species.

Species	Sequence data	Nucleotide sequence identity (%)	Amino acid sequence identity(%)
Goat	XM_005684148.2	98.82	98.39
Bovine	NM_001076439.2	96.14	93.61
Porcine	NM_001244790.1	89.14	83.18
Cat	XM_003995460.5	86.71	83.81
Dog	XM_025423094.1	85.32	81.62
Human	NM_000197.2	84.46	79.13
Monkey	NM_001266504.2	84.46	77.88
Donkey	XM_014856940.1	83.90	78.62
Horse	XM_023627093.1	80.41	73.91
Rabbit	XM_002708264.3	79.53	73.56
Rat	NM_054007.1	77.93	71.12
Mouse	NM_008291.3	77.72	71.87
Chicken	XM_425046.6	66.49	57.86
